# Modelling Eddy Current Testing of Gaps in Carbon Fibre Structures Based on Spline Approximation

**DOI:** 10.3390/s26031032

**Published:** 2026-02-05

**Authors:** Till Schulze, Maren Rake, Dirk Hofmann, Johannes Mersch, Martin Schulze, Chokri Cherif, Henning Heuer

**Affiliations:** 1 Fraunhofer Institute for Ceramic Technologies and Systems IKTS, 01109 Dresden, Germany; 2 Institute of Electronic Packaging Technology and Centre for Microtechnical Manufacturing, Technical University Dresden, 01069 Dresden, Germany; 3 Institute of Textile Machinery and High Performance Material Technology, Technical University Dresden, 01069 Dresden, Germany

**Keywords:** eddy current testing, carbon fibre, computational electromagnetics, FEM, anisotropic conductivity, gaps

## Abstract

Defects such as gaps, delamination, and the misalignment of fibres impair the performance of carbon fibre-reinforced composites and can lead to structural failure during operation. Eddy current testing has proven to be a suitable method for detecting these defects early in the manufacturing process. However, validated electromagnetic modelling techniques are required to develop new eddy current sensors and gain a better understanding of the eddy current signals caused by different defect sizes. This paper proposes a novel finite element modelling approach to better account for fibre heterogeneity using spline approximation. Further, adaptive mesh refinement is used to reduce FEM solution errors. A defect in the form of a gap is modelled by adjusting the spline approximation accordingly. Finally, the model also accounts for inter-laminar current paths between carbon fibre layers, which are determined by four-terminal resistance measurement. The results show that the electromagnetic properties of the structure can be successfully modelled. The simulation is validated by comparing the virtual scans with eddy current scans of dry carbon fibre fabric with and without artificially manufactured gaps.

## 1. Introduction

The inline production monitoring of multiaxial carbon fibre (CF) fabrics requires the inspection of non-visible hidden layers, preferably by means of non-destructive testing. Eddy current testing has proven to be particularly suitable for this purpose [1,2]. A newly developed system at Fraunhofer IKTS is using eddy current probe array technology to provide information about fibre orientation, gaps, undulations, ply build-up, the inclusion of foreign materials, fibre fuzz and splices in visible and hidden layers [3].

**Figure 1:**
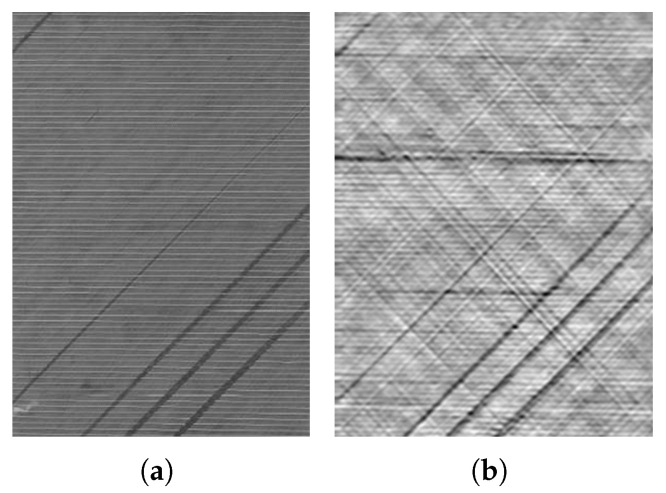
Three-layer CF fabric with missing fibre bundles: photography (**a**) and EC scan (**b**) [9].

Due to the complex physical effects underlying the eddy current method, especially at high frequencies, and the electrically complex properties of CF structures [4,5,6], the interaction between CF materials and electromagnetic fields in terms of eddy current density distribution and the resulting effects on the electrical behaviour of a coil is still subject to current research [7,8]. This makes it difficult to accurately interpret defect dimensions. Nonetheless, the process of inline inspection demands the precise measurement of defect sizes within the fabric. It is imperative that the propagation of eddy currents in carbon fibre layers with missing fibre bundles is thoroughly understood to successfully complete this task. An illustration of such gaps in a fabric is provided in Figure 1, together with the corresponding eddy current scan.

The objective of this study is to propose innovative methodologies for simulating defects in carbon fibres characterised by known conductivity, with a view to enhancing the comprehension of eddy current defect signals. However, advances in finite element method (FEM) simulation have also facilitated the development of novel eddy current sensor designs. The ability to engineer coils that demonstrate a responsiveness to alterations in conductivity within new materials or for specific applications, without being solely contingent on experimentation and experience, is a significant advancement. Finally, it is imperative to employ precise modelling techniques to generate authentic simulated eddy current data. This is essential for accumulating sufficient training data for machine learning algorithms in automatic defect characterisation.

In the context of anisotropic and heterogeneous materials, such as carbon fibre fabrics, modelling the structure is of crucial importance and contributes to the accuracy of the FEM simulation. The heterogeneity of the fibres is attributable to the higher conductivity observed in the centre of a roving relative to its edge area. This paper proposes a novel methodology founded upon the premise of spline approximation, with the objective of modelling the conductivities within the respective CF layers. This approach is also employed in the modelling of defects in the fabric. Further, the simulation accounts for interlaminar current paths between carbon-fibre layers that are determined in resistance measurements. The simulation is validated by comparing the simulated scans with eddy current scans of dry carbon-fibre fabric, including configurations with artificially introduced gaps and intact samples. This paper analyses dry fabric samples to minimise the matrix’s influence and focus on the fibres, as eddy current inspection is especially beneficial early in CFRP production. It monitors dry fabrics for which ultrasonic testing is limited by poor coupling.

## 2. State-of-the-Art

### 2.1. Eddy Current Simulation—General Parameters

Unlike other scientific disciplines, such as mechanics, electromagnetic simulation requires the air surrounding a test object to be meshed. This results in the area being divided into a non-conductive and a conductive region [10]. In FEM simulation eddy current sensors are modelled as hollow cylinders. Modelling individual turns would require a considerable amount of computing time, which is not justified by significantly more accurate results [11].

### 2.2. Eddy Current Simulation—CF Parameters

**Figure 2:**
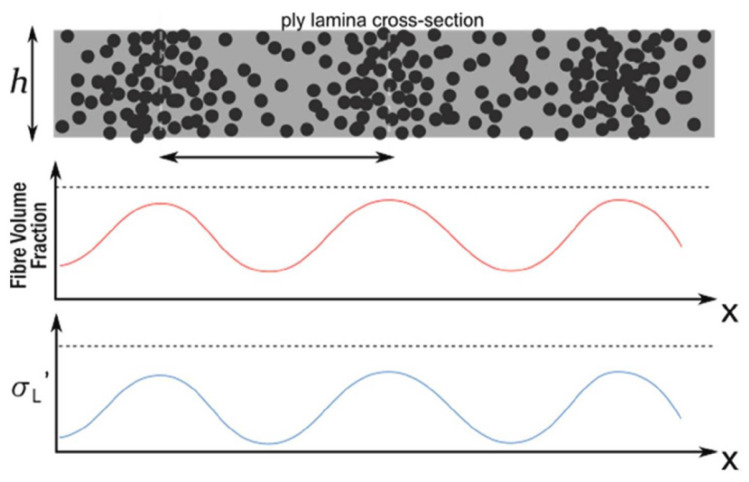
Proposed principle for simulated conductivity changes in a unidirectional CFRP layer according to [14].

Compared to isotropic materials, no constant value can be assumed for the conductivity of CFRP. In addition to the direction-dependent properties, the heterogeneity resulting from the structure must also be considered. Furthermore, due to the thin layers, fine cross-linking is required, taking into account the conditions between the layers. The latter was investigated in [7] (skin effect at different layers with varying orientation). The tensor for conductivity in the mapping of anisotropic properties as a function of the fibre angle, $\theta_{F}$, can be introduced as follows [12]:

(1) $$\begin{array}{c} \sigma =\left[\begin{array}{ccc} \sigma_{L}\cos^{2}\theta_{F}+\sigma_{T}\sin^{2}\theta_{F} & \frac{\sigma_{L}-\sigma_{T}}{2}sin2\theta_{F} & 0\\ \frac{\sigma_{L}-\sigma_{T}}{2}sin2\theta_{F} & \sigma_{L}\sin^{2}\theta_{F}+\sigma_{T}\cos^{2}\theta_{F} & 0\\ 0 & 0 & \sigma_{cp} \end{array}\right] \end{array}$$

where $\sigma_{L}$ is the longitudinal conductivity in the fibre direction, and $\sigma_{T}$ is the conductivity transverse to it, while $\sigma_{cp}$ describes the conductivity in through-thickness direction. Most researchers use Equation (1) as a homogenised conductivity tensor to model CFRP materials, which fails to model the inhomogeneities resulting from the material structure [13]. The latest research on simulating eddy current testing in composites by Yi et. al. is considering the heterogeneity of the layers [14]. For this purpose, location-dependent functions for conductivity were established instead of constant factors as in [5,15,16,17] in the form of Equation (2) and as visualised in Figure 2.

(2) $$\begin{array}{c} f\left(x\right)=a\cdot sin(\frac{2\pi }{b}\cdot \left(x\right))+c \end{array}$$

where a, b and c are constants to approximate the conductivity regarding the amplitude and periodicity of the fibres, and f(x) is considered the fibre volume fraction. The formulation for the effective conductivity at a given location is combining Equations (1) and (2) to

(3) $$\begin{array}{c} \sigma_{ij}^{\prime }\left(x,\theta_{F}\right)=\sigma_{ij}\left(\theta_{F}\right)\cdot f\left(x\right) \end{array}$$

Finally, the conductivity function is extended to two-dimensional space by varying f(x), depending on x and y position, which results in $\sigma_{ij}^{\prime }\left(x,y,\theta_{F}\right)$. Yi’s work [14] is also considering resin-rich areas, which can be neglected in this study, as dry fabrics are modelled.

The concept of virtual scanning, coined in Yi’s work [14], can be used to significantly reduce processing times while also decreasing numerical noise. Moving the sensor over the structure or moving a gap in the structure results in a re-meshing of the model with each iteration. Instead, virtual scanning fixes the geometry and position of the coil and material and only changes the offset of the coordinates $x_{n}$ and $y_{m}$ in the conductivity parameter space $\sigma_{ij}^{\prime }\left(x+x_{n},y+y_{m},\theta_{F}\right)$ [14].

## 3. New Modelling Approach

**Figure 3:**
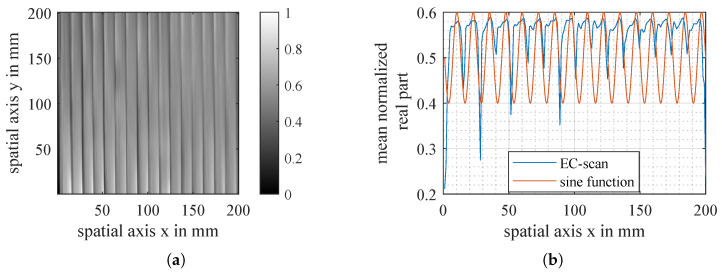
EC-scan of unidirectional carbon fibre fabric (**a**) and mean value with fitted sine function (**b**).

The approach of Yi is the first attempt to model the heterogeneity of conductivity in carbon fibres. An example of an eddy current scan of unidirectional CF fabric in Figure 3 shows that simple sine functions do not approximate the real behaviour of fibres with sufficient accuracy. In fact, the space between rovings and, therefore, the area of lower conductivity is much shorter than simple sine functions can represent.

In the middle of a roving, its conductivity reaches its maximum due to high fibre packing density leading to more internal contacts and, therefore, more conduction paths. Close to the borders of a roving, the package density is lower, leading to less stable contacts and lower conductivity. The biggest drop in conductivity happens in between rovings, where contacts are few, small voids occur, and the pressure between rovings is low [18]. The described mechanism and observed pattern that can be seen in Figure 3 were also measured in [19]. To describe this behaviour, this paper proposes a piecewise defined function—a constant conductivity inside the core up to a defined radius, $r_{0}$, followed by a slow decrease that transitions into an abrupt change at the contact point with the next roving, which is the roving radius R:

(4) $$\begin{array}{c} \sigma_{L}\left(r\right)=\left\{\begin{array}{cc} \sigma_{L}, & 0\leq r\leq r_{0}\\ -\frac{\alpha }{r+\beta }+\gamma , & r_{0}\leq r\leq R \end{array}\right. \end{array}$$

The parameter α defines the smoothness of the transition between high and low changes in the conductivity curve. In this way, the function can be flexibly adapted to different filament counts, diameters and distances between them. To simplify the calculations of the offsets β and γ, a conductivity of 0 S/m at radius *R* and a conductivity of 1 S/m at radius r=0 is defined. Then, the equations are

(5) $$\begin{array}{c} \beta =\frac{1}{2}r_{0}\left(\sqrt{\frac{4\alpha }{r_{0}}+1}-1\right)\;\mathrm{and}\;\gamma =\frac{\alpha }{\beta } \end{array}$$

With Equations (4) and (5) it is possible to calculate values at defined points and rescale them to achieve a conductivity distribution of a roving. This is replicated for multiple rovings to achieve a position-dependent conductivity distribution for the whole carbon fibre layer. To implement these pairs of values into the simulation software, a piecewise approach based on a defined polynomial function is introduced. Therefore, this paper proposes a conductivity function using a linear spline approximation to replace Equation (2) by Equation (6).

(6) $$\begin{array}{c} f\left(x\right)=\sigma_{0}+m_{0}\left(x-x_{0}\right)+\sum_{i=1}^{N-2}\left(m_{i}-m_{i-1}\right)\cdot \frac{\left(x-x_{i}\right)+\left|x-xi\right|}{2} \end{array}$$

(7) $$\begin{array}{c} \;\mathrm{with}\;m_{i}=\frac{\sigma_{i+1}-\sigma_{i}}{x_{i+1}-x_{i}},\;\;i=0,\ldots ,N-2 \end{array}$$

**Figure 4:**
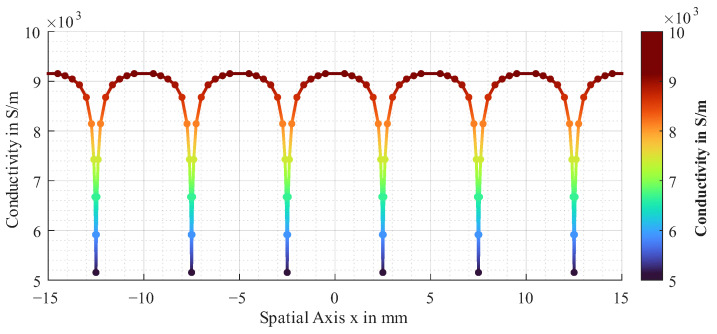
Approximated function for electrical conductivity of unidirectional CF layer.

With *x* being the spatial coordinate transverse to the fibres and $\sigma_{i}$ the conductivity to which the spline is approximated for a more precise representation of the heterogeneity. The proposed approximated function for the electrical conductivity is shown in Figure 4.

It represents the spline, which is approximated by the points in the plot with N=286.

**Figure 5:**
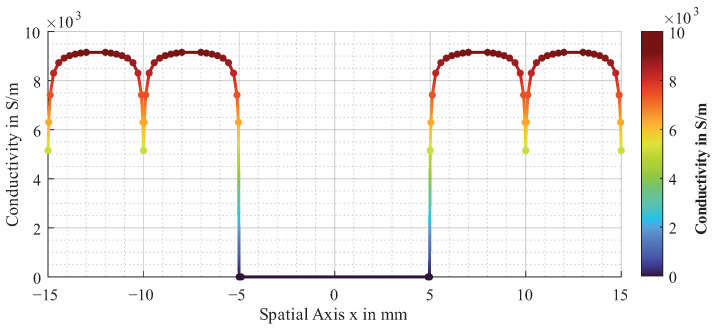
Approximated function for electrical conductivity of unidirectional CF layer with gap.

If this approach is pursued further, defects can also be described in terms of conductivity. This paper primarily focuses on gaps. Gaps are missing fibre bundles and can, therefore, be described as areas with a conductivity of approximately 0 S/m. This behaviour can also be approximated using splines, as shown in Figure 5. It is essential that there is a sufficient ”normal” area, i.e., defect-free material, to the right and left of the gap.

## 4. Sample Preparation and Experimental Setup

### 4.1. CF Samples

**Figure 6:**
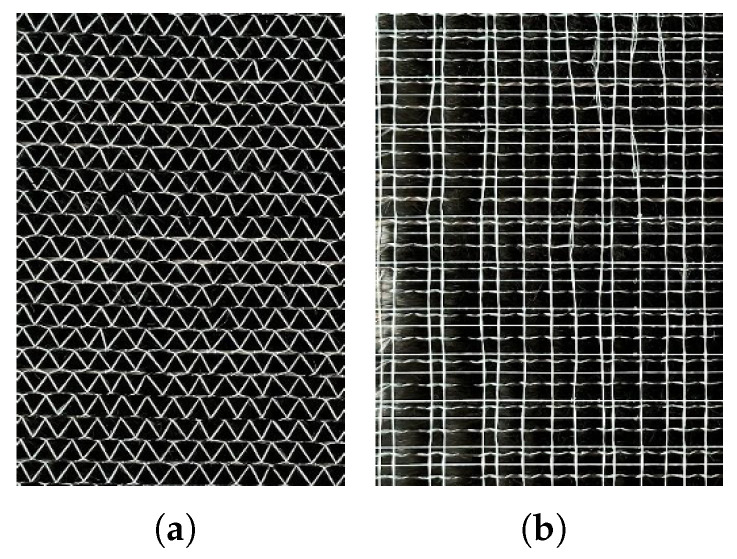
Unidirectional carbon fibre fabric front (**a**) and back (**b**).

Single-layer unidirectional (UD) fabrics with a weight of 300 g/m^2^ are used to produce the multi-layer test samples used in this study. The roving width is 5 mm, with each roving containing 50 k fibres. Figure 6 shows such a UD fabric from the front and back, and it has been stitched with glass fibre yarn.

**Figure 7:**
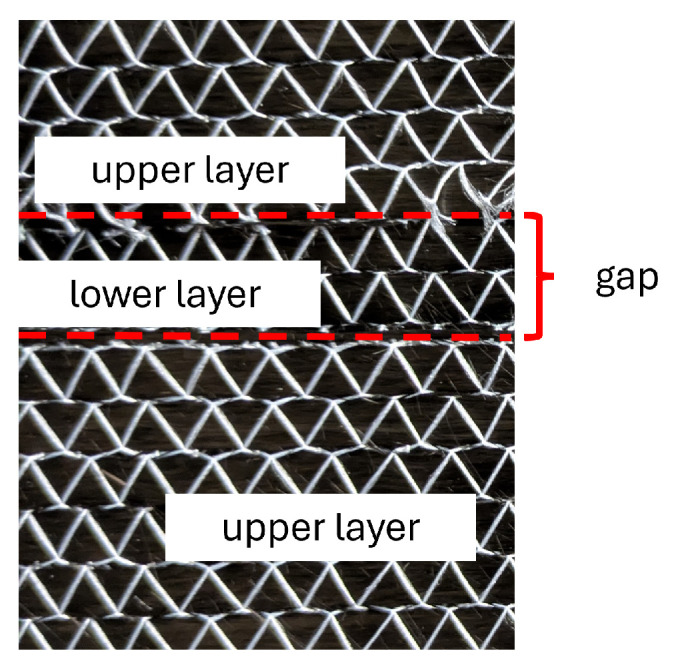
Creating a gap in fabric.

Multi-layer samples are made by assembling several UD-samples of a size of 20 × 20 cm^2^ with different orientations. A gap is artificially manufactured by cutting one ply and leaving a gap of 10 mm between both fabric segments, as shown in Figure 7.

### 4.2. Experimental Setup

**Figure 8:**
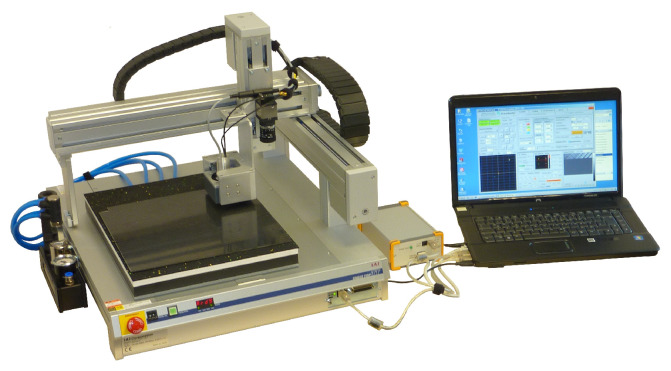
Eddy current testing setup - MPECS with Analysis PC [20].

All measurements were performed using the EddyCus^®^ Multi Parameter Eddy Current Scanner (MPECS) by Fraunhofer IKTS, Dresden, Germany visualised in Figure 8 [20].

The table-top robot scans with a 0.23 mm step size in the x-direction and a 1 mm step size in the y-direction over a 100 × 100 mm^2^ area in the middle of the samples. As the edges of the sample cause a significant change in the eddy current signal, that shall not be investigated in this study. A spring-loaded probe mount and a protection tape of 0.08 mm ensure constant lift-off. The Tx-Rx-coil is operated in reflection mode and has relatively low inductance with 30 turns on the sending coil and 50 turns on the receiving coil. Both coils are inserted in pot cores at a distance of 2.84 mm. A preamplifier is mounted directly behind the coils to eliminate disturbing high-frequency effects caused by the connection cables. A Tx-Rx coil is chosen because studies by Mook and Schmidt [21,22] have shown that this type of coil has a significantly higher resolution for carbon fibre materials.

## 5. FE Modelling

### 5.1. Interface Resistivity

According to Cheng et al. [15], the inter-laminar current paths between layers with different fibre orientations lead to an increase in the eddy current density in the boundary layer between them. The greater the angular offset between the layers, the higher the induced eddy currents at the interface [15].

The eddy current density at the interface between layers can even be higher than that at the surface of the material, which is why local defects in this area can cause stronger measurement signals than defects near the surface [23].

**Figure 9:**
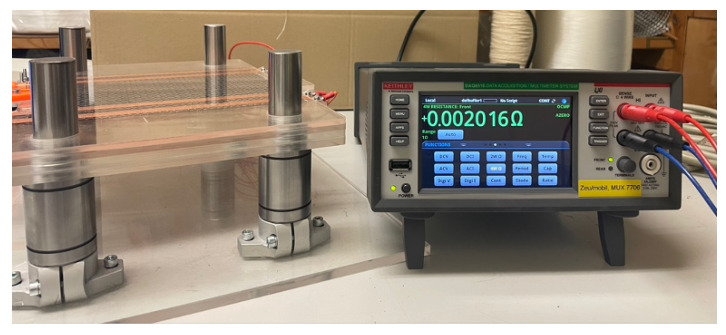
Experimental setup for resistance measurement.

To test this theory and determine the values of the interface resistances between layers for use in the simulation, a four-terminal sensing method for different layer structures on dry fabric has been applied. The experimental setup for electrical resistance measurement is shown in Figure 9. The electrode strips are each 12 mm wide. The distance between the middle electrodes is 115 mm. A total weight of 1 kg was used on top of the CF-layers to apply reproducible pressure. The resistance was measured with a Keithley DAQ6510 digital multimeter by Keithley Instruments, Solon, OH, USA and a four-terminal method was used to minimise errors from lead and terminal resistances.

In each case, the resistance was determined at the interface between layers, which is separated from the other layers by a plus sign in Table 1. The results confirm the theory that a large angular offset between layers and a high number of layers reduces the interface resistance and thus increases the potential eddy current density.

### 5.2. FEM Setup

**Figure 10:**
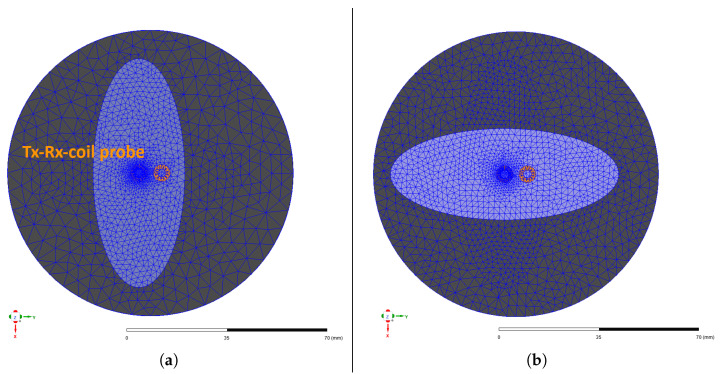
Illustration of FEM meshing geometry in ANSYS Maxwell for 0° (**a**) and 90° (**b**) layer orientation.

**Figure 11:**
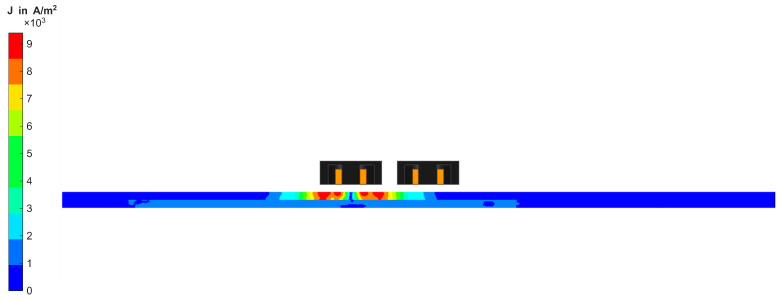
Cross section of Tx-Rx probe with pot cores in FEM simulation.

As shown in Figure 10, the CF domain is a cylindrical volume with a radius of 30 mm, representing the area of the dry CF fabric around the semi-transmission Tx-Rx-coil probe. Since the eddy currents in the material primarily propagate in an elliptical pattern in the direction of the fibres [23,24], an elliptical area below the coil was provided with a finer mesh in each fibre direction. This kind of adaptive mesh refinement is used to reduce the error of the FEM solution, which stems from discretisation, leading to discrepancies between the computed and true physical solution. The Maxwell solver increases the local element density by about 30% between successive iterations to reduce the change in the receiver coil’s induced voltage and thus improve convergence. The CF domain contains ten fibre bundles in 0° and 90° orientations, modelled by the conductivity function introduced in Section 3. A small area is used to simulate virtual scanning to reduce the required computing time and resources. Further parameters of the model are summarised in Table 2. The conductivities used for simulation have been determined experimentally via the four-terminal sensing method, in the same way as for interface resistivity measurement. The interface resistivity is simulated by assigning a resistive sheet boundary to the contacting surface of both layers. The probe was modelled to correspond with the same conditions present in the experiment, as can be seen in Figure 11. In a second modelling process, two adjacent fibre bundles were removed from the CF domain to simulate a gap. The gap is equally introduced by the conductivity function introduced in Section 3.

## 6. Results and Discussion

### 6.1. Modelling Results

**Figure 12:**
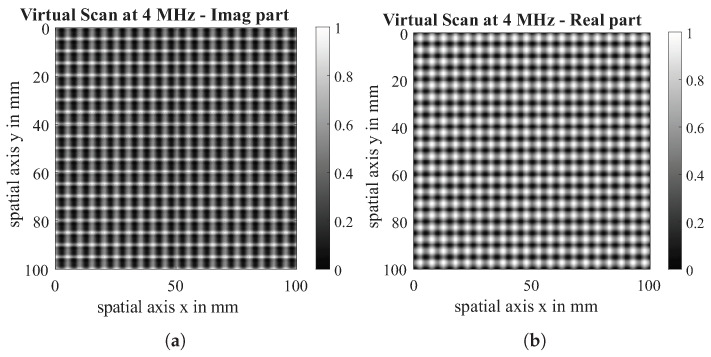
Virtual scan of 0|90 dry CF fabric: imaginary part (**a**) and real part (**b**) at a measuring frequency of 4 MHz.

Simulated 2D ECT Scans are presented in Figure 12. These virtual scans are the result of scanning a 0|90 gap-free dry carbon fibre fabric with the parameters shown in Section 5.2. In order to compare simulation and test data and ensure comparability with Yi’s results [14], both the real part and the imaginary part are scaled to a value between (0, 1). Additionally, no further processing of amplitude or phase is used for the same reasons. Since a Tx-Rx coil is used and is aligned parallel to the 90° layer orientation, this layer is represented more strongly than the 0°-layer.

### 6.2. Validation

**Figure 13:**
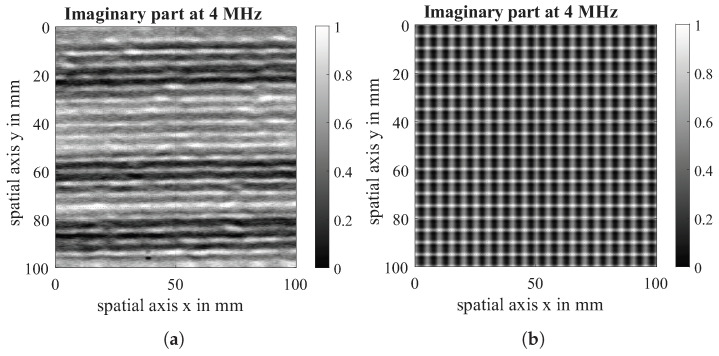
Imaginary part of EC-scan of 0|90 fabric with 10 mm gap (**a**) and imaginary part of virtual Scan of simulated gap (**b**) at a measuring frequency of 4 MHz.

**Figure 14:**
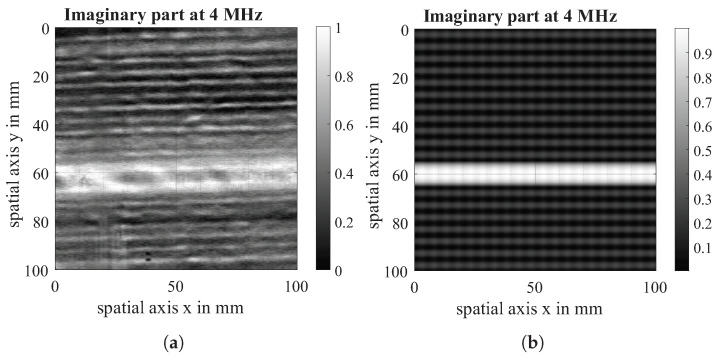
Real part of EC-scan of 0|90 fabric (**a**) and real part of virtual scan (**b**) at a measuring frequency of 4 MHz.

**Figure 15:**
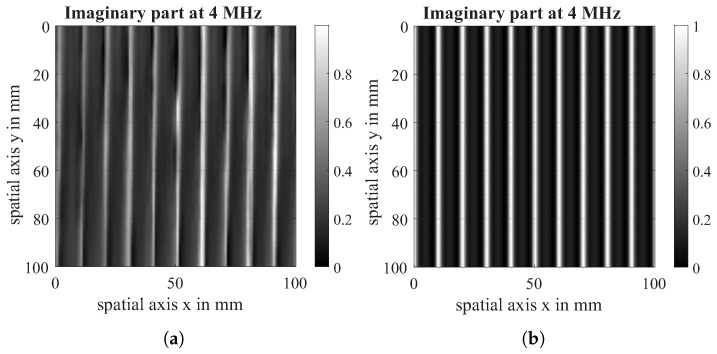
Imaginary part of EC-scan of 0° unidirectional fabric (**a**) and imaginary part of virtual Scan (**b**) at a measuring frequency of 4 MHz.

The imaginary part of the virtual scan is compared with the imaginary part of the eddy current scan both at a measurement frequency of 4 MHz. The data acquired in the experiment is visualised on the left of Figure 13, Figure 14 and Figure 15 while the data from the simulation can be seen on the right of all figures. For comparability, the experimental dataset has been scaled likewise to a value between (0, 1), and the contrast of the raw data has been enhanced. It is visible that the defect shows the same behaviour in simulation and experiment. The direction-dependent response behaviour of the Tx-Rx coil is visible in both the simulation and the experiment. The 90°-layer is represented more strongly in both cases. Further, the experiments with 0|90 fabric show spatial variation across the samples, which is due to spatial fluctuations in conductivity and permittivity caused by variations of fibre density and contact between and within fibre bundles that are not reflected in the simulation. Future research work will further analyse these material properties and integrate them into the conductivity function.

Finally, a unidirectional 0°-layer was modelled to assess the similarity of the conductivity function with the real material behaviour, which can be seen in Figure 15.

**Figure 16:**
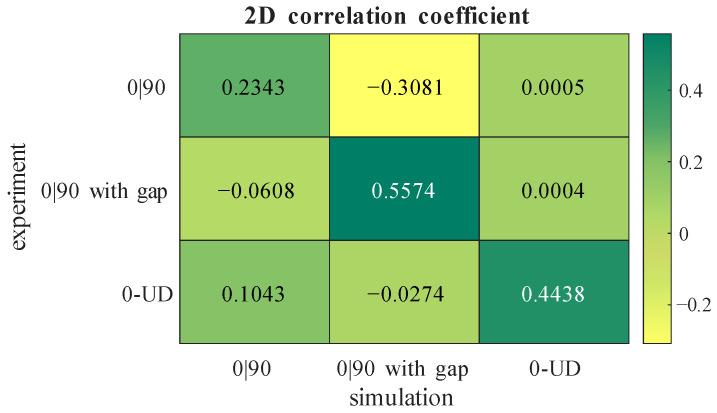
2D correlation coefficient between simulation and experiment.

With the use of a 2D correlation coefficient (corr2()-matlab function, Matlab 2025b), the similarity between experiment and simulation is quantitatively evaluated. The results are shown in Figure 16 in the form of a confusion matrix between each experimental and simulated result for the imaginary parts of the measured voltage. For every case, the correlation is strongest when the simulation’s layup matches the experimental layup. The comparison between the simulation and the experiment with the gap, shows the highest correlation. The correlation between the experiment and the simulation of 0|90 fabric is lower than that of the others. This can be explained by the eddy current scan, which shows a very low influence of the 0°-layer compared to the 90°-layer due to the sensor orientation. In the simulation, this influence is not that high, and it needs to be investigated in future simulations.

## 7. Conclusions and Future Work

This paper has presented a novel approach to simulating eddy current testing in carbon fibres. On the one hand, representing the heterogeneity of fibres with spline approximation offers the user a high degree of flexibility regarding fibre shape and size while, at the same time, enabling a more realistic simulation of their behaviour. In addition, the user can insert almost any type of defect with known conductivity without having to model it. Subsequent investigations will concentrate on enhancing the model’s precision by incorporating statistical material variations. A future objective is to characterise several gap widths in different fabric layers.

## Figures and Tables

**Table 1:** Measured resistance at the interface of different carbon fibre fabric layers.

CF-Layers	Interface Resistance in Ω
0° (300 g/m^2^) + 0° (300 g/m^2^)	131.52
0° (300 g/m^2^) + 90° (300 g/m^2^)	92.38
0° (500 g/m^2^) + 0° (500 g/m^2^)	12.13
0° (500 g/m^2^) + 90° (500 g/m^2^)	12.05
0° (1000 g/m^2^) + 0° (1000 g/m^2^)	7.27
0°|90°|45°|45° (1000 g/m^2^) + 0° (1000 g)	5.40
0°|90° (1000 g/m^2^) + 0° (1000 g/m^2^)	5.25
0° (1000 g/m^2^) + 90° (1000 g/m^2^)	4.73
0°|90°|90°|0° (1000 g/m^2^) + 90° (1000 g/m^2^)	3.53
0°|90°|45°|45° (1000 g/m^2^) + 90° (1000 g/m^2^)	3.33
0°|90°|90°|0° (1000 g/m^2^) + 0° (1000 g/m^2^)	3.28
0°|90° (1000 g/m^2^) + 90° (1000 g/m^2^)	3.01
0°|90°|45°|45°|45°|45°|90°|0° (1000 g/m^2^) + 90° (1000 g/m^2^)	2.69
0°|90°|45°|45°|45°|45°|90°|0° (1000 g/m^2^) + 0° (1000 g/m^2^)	2.65

**Table 2:** Modelling parameters.

Domain	Parameter	Value
Sensor (represented as two identical coils)	Coil distance	2.8 mm
	Inner Coil radius	0.5 mm
	Outer Coil radius	0.75 mm
	Coil height	1.0 mm
	Windings Transmitter	30
	Windings Receiver	50
	Frequency	4 MHz
	Core Material	F10b
	Lift-Off	0.5 mm
CF Laminate (modelled as 2 circular layers)	$\sigma_{L}$,	5154–9154,
	$\sigma_{T}$, $\sigma_{cp}$	40.5, 10 S/m
	layer thickness	0.6 mm
Simulation Parameters	Sofware	Ansys Maxwell 2025 R2
	Solution Type	Eddy Current
	α from Equation (5)	0.1
	$r_{0}$ from Equation (5)	2
	*N* from Equation (6)	286

## Data Availability

The data presented in this study are available on request from the corresponding author.

## Abbreviations

The following abbreviations are used in this manuscript:

CF	Carbon fibre
EC	Eddy current
ECT	Eddy current testing
FEM	Finite Element Method
MPECS	Multi Parameter Eddy Current Scanner
Rx	Receiver
Tx	Transmitter
UD	Unidirectional

## References

[1] Bardl G., Kupke R., Heuer H., Cherif C. (2018). Eddy current testing in CFRP production. Lightweight Des. Worldw..

[2] Heuer H., Schulze M., Pooch M., Gäbler S., Nocke A., Bardl G., Cherif C., Klein M., Kupke R., Vetter R. (2015). Review on quality assurance along the CFRP value chain—Non-destructive testing of fabrics, preforms and CFRP by HF radio wave techniques. Compos. Part B Eng..

[3] Schulze M. (2023). Full-Volume Quality Control and Online Evaluation of Carbon Fiber Fabrics. Annual Report 2022/23. https://epaper.ikts.fraunhofer.de/epaper-fraunhofer-ikts-annual-report-2022-23/#1.

[4] Wang T., Wu D. (2023). A modified conductive network used to characterize the conductivity of carbon fibre reinforced polymers in eddy current testing. Compos. Struct..

[5] Bui H.K., Wasselynck G., Trichet D., Berthiau G. (2016). Degenerated Hexahedral Whitney Elements for Electromagnetic Fields Computation in Multi-Layer Anisotropic Thin Regions. IEEE Trans. Magn..

[6] Horie T., Miyake R., Tanaka Y., Kamihara N., Matsuyama D., Takagi K., Muraoka M. (2024). Resistivity model of carbon fiber cloth considering the effect of interlayer contact resistance. Compos. Part A Appl. Sci. Manuf..

[7] Xu X., Dai T., Luo J., Yan Y., Qiu J. (2023). Propagation characteristics of electromagnetic field penetrated into laminated CFRPs using eddy current testing with pancake coil. Compos. Struct..

[8] Hughes R., Drinkwater B., Smith R. (2018). Characterisation of carbon fibre-reinforced polymer composites through radon-transform analysis of complex eddy-current data. Compos. Part B Eng..

[9] Schulze M., Küttner M., Heuer H., Meyendorf N. (2009). Mehrfrequenz-Wirbelstromprüfverfahren zur Qualitätskontrolle bei der Produktion von Kohlefaser-Multiaxialgelegen.

[10] Silvester P.P. (1996). Finite Elements for Electrical Engineers.

[11] Bossavit A., Mayergoyz I.D. (1998). Computational Electromagnetism Variational Formulations, Complementarity, Edge Elements.

[12] Cheng J., Ji H., Qiu J., Takagi T., Uchimoto T., Hu N. (2014). Role of interlaminar interface on bulk conductivity and electrical anisotropy of CFRP laminates measured by eddy current method. NDT E Int..

[13] Cheng J., Wang B., Xu D., Qiu J., Takagi T. (2021). Resistive loss considerations in the finite element analysis of eddy current attenuation in anisotropic conductive composites. NDT E Int..

[14] Yi Q., Wilcox P., Hughes R. (2023). Modelling and evaluation of carbon fibre composite structures using high-frequency eddy current imaging. Compos. Part B Eng..

[15] Cheng J., Ji H., Qiu J., Takagi T., Uchimoto T., Hu N. (2014). Novel electromagnetic modeling approach of carbon fiber-reinforced polymer laminate for calculation of eddy currents and eddy current testing signals. J. Compos. Mater..

[16] Cheng J., Qiu J., Ji H., Wang E., Takagi T., Uchimoto T. (2017). Application of low frequency ECT method in noncontact detection and visualization of CFRP material. Compos. Part B Eng..

[17] Menana H., Feliachi M. (2011). An Integro-Differential Model for 3-D Eddy Current Computation in Carbon Fiber Reinforced Polymer Composites. IEEE Trans. Magn..

[18] Athanasopoulos N., Sikoutris D., Siakavellas N., Kostopoulos V. (2015). Electrical resistivity prediction of dry carbon fiber media as a function of thickness and fiber volume fraction combining empirical and analytical formulas. Compos. Part B Eng..

[19] Buser Y., Krämer E., Akkerman R., Grouve W. (2025). Reliable longitudinal electrical conductivity characterisation of unidirectional CFRP tapes. Compos. Part A Appl. Sci. Manuf..

[20] Heuer H., Schulze M., Klein M. (2012). 5.2.3 Abbildende Wirbelstromsensoren zur hochauflösenden berührungslosen Abbildung von elektrischen Eigenschaften schlecht leitender Objekte. Proceedings of the Tagungsband.

[21] Mook G., Michel F., Simonin J. (2011). Electromagnetic Imaging Using Probe Arrays. Stroj. Vestn. J. Mech. Eng..

[22] Schmidt C., Schultz C., Weber P., Denkena B. (2014). Evaluation of eddy current testing for quality assurance and process monitoring of automated fiber placement. Compos. Part B Eng..

[23] Bardl G. (2019). Entwicklung Eines Verfahrens Zur Zerstörungsfreien Messung der Faserorientierung in Mehrlagigen 3D-Carbonfaserpreforms und CFK Mit Robotergeführter Hochfrequenz-Wirbelstromprüftechnik. Ph.D. Thesis.

[24] Mook G., Lange R., Koeser O. (2001). Non-destructive characterisation of carbon-fibre-reinforced plastics by means of eddy-currents. Compos. Sci. Technol..

